# Appropriate hinge position to prevent hinge fracture in open wedge distal tuberosity tibial osteotomy

**DOI:** 10.1002/jeo2.70278

**Published:** 2025-05-19

**Authors:** Atsuki Tanaka, Daisuke Araki, Takahiro Yamashita, Shohei Sano, Ryo Okada, Mitsuhiko Takahashi, Yasushi Hashimoto

**Affiliations:** ^1^ Department of Orthopaedic Surgery Hyogo Prefectural Rehabilitation Central Hospital Kobe Japan; ^2^ Department of Orthopaedic Surgery Kobe University Graduate School of Medicine Kobe Japan; ^3^ Sports Medicine Center Hyogo Prefectural Rehabilitation Central Hospital Kobe Japan

**Keywords:** hinge fracture, hinge position, open wedge distal tuberosity tibial osteotomy, open wedge high tibial osteotomy, varus knee osteoarthritis

## Abstract

**Purpose:**

Open wedge distal tuberosity tibial osteotomy (owDTO) is an effective treatment for varus knee osteoarthritis. Hinge fracture is a major complication, and hinge position is a contributing factor. This study aimed to investigate the relationship between hinge position and fractures.

**Methods:**

This study examined 42 knees that underwent owDTO. The level of the proximal tibiofibular joint (PTFJ) hinge fracture was investigated on postoperative computed tomography (CT) images measuring the distances and angles around hinge area. Based on previous reports, the hinge position was classified, and hinge fractures were assessed.

**Results:**

No cases were found with a hinge position above PTFJ, 41 cases were within PTFJ, and one case was distal to PTFJ. Hinge fractures were observed in 10 patients (23.8%). According to the hinge position classification, seven cases were type I (a fracture within PTFJ), three were type II (a fracture that reaches the distal portion of PTFJ), and no cases were type III (intra‐articular fracture). The anteroposterior hinge width measured perpendicular to the descending cut of the flange on axial CT images at the proximal level of the PTFJ was significantly shorter in the hinge fracture group (*p* = 0.03).

**Discussion:**

The owDTO hinge fracture rate in our study was comparable to that in previous reports on open wedge high tibial osteotomy. As the risk of hinge fracture increases with the shortening of the anterior‐posterior hinge width, our results indicate that preservation of the anterior‐posterior hinge width may help reduce hinge fractures.

**Level of Evidence:**

Level III, Retrospective study.

**Abbreviations**
CT: computed tomography
HTO: high tibial osteotomy
mMPTA: mechanical medial proximal tibial angle
owDTO: open wedge distal tuberosity tibial osteotomy
owHTO: open wedge high tibial osteotomy
PTFJ: proximal tibiofibular joint
ROC: receiver operating characteristic
%MA: percentages of mechanical axis

## INTRODUCTION

High tibial osteotomy (HTO) is an effective treatment for varus knee osteoarthritis [7, 8, 15, 22, 24]. Moreover, HTO preserves the original joint function, unlike total knee arthroplasty [22]. This approach has been reported to be effective for knee osteoarthritis but has received little attention due to difficulties in early weight‐bearing [15]. The development of locking plates has improved stability, allowing for early weight‐bearing [5, 23, 24].

HTO involves techniques such as closed wedge, dome, and open wedge [15]. Although open wedge HTO (owHTO) is widely performed, complications such as hinge fracture, neurovascular injury, wound infections, osteoarthritis of the patellofemoral joint, irritation from the implant, and compartment syndrome can occur [26]. Specifically, it is important to prevent hinge fractures because they could cause delayed bone union or bone malunion [19].

**Figure 1:**
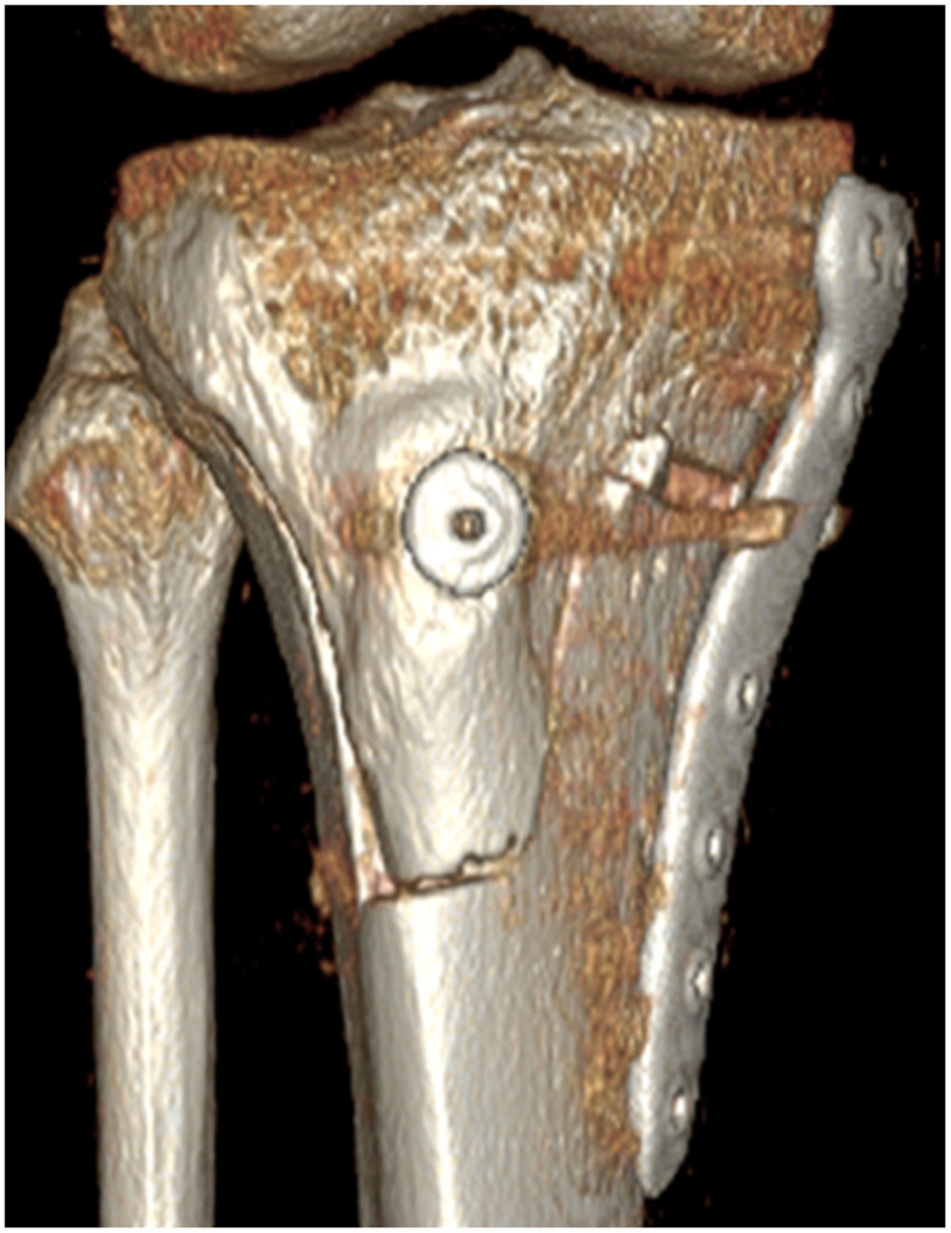
Postoperative computed tomography of open wedge distal tuberosity tibial osteotomy.

Open wedge distal tuberosity tibial osteotomy (owDTO), which is one of types of owHTO, is a technique that leaves the tibial tuberosity attached to the proximal bone fragment, with the advantage of preventing increased contact pressure on the patellofemoral joint, which is a concern in conventional owHTO (Figure 1) [1]. Therefore, the hinge width of owDTO might be narrower by the thickness of the flange than owHTO because the osteotomy way is different from that of owHTO. Han et al. examined the hinge fractures of owHTO in terms of the hinge position, opening width, and technical errors during operation [6]. However, to the best of our knowledge, no clinical reports have examined the factors contributing to hinge fractures in owDTO. We hypothesised that appropriate hinge positioning would result in the rate of hinge fracture comparable to owHTO. This study aimed to investigate the relationship between the hinge position and fracture in owDTO.

## METHODS

### Patient selection

This retrospective study was performed in accordance with the Declaration of Helsinki and approved by our institutional review board. All patients were informed that they were going to be included in a study. Fifty knees of 46 patients (22 male and 24 female patients) who underwent owDTO at our institution between May 2021 and May 2022 were examined. Patients who underwent postoperative follow‐up at a different hospital or who had not undergone computed tomography (CT) within 3 weeks after surgery were excluded. After patient exclusion, our analysis included 42 knees in 38 patients (17 male and 21 female patients with an average age of 55.0 ± 10.6 years (minimum: 32 years and maximum: 79 years) and an average body mass index of 25.4 ± 3.3 kg/m^2^ (minimum: 18.7 kg/m^2^ and maximum: 32.3 kg/m^2^). The follow‐up rate was 84% (42/50 knees). The follow‐up period ranged from 22 to 33 months (27.0 ± 3.0 months).

### Surgical treatment

**Figure 2:**
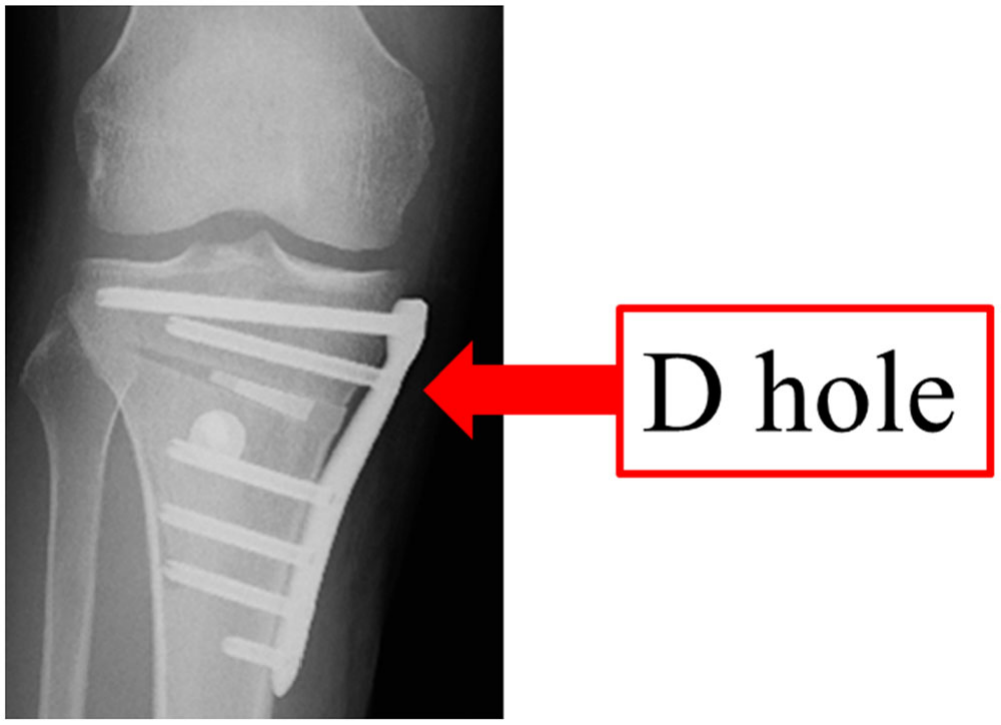
Postoperative radiography of open wedge distal tuberosity tibial osteotomy.

All patients underwent owDTO according to the method described by Akiyama et al. [1]. The hinge location was determined by setting the osteotomy line to allow the maximum length screw possible (60 mm) to be inserted into the D‐hole of the plate (Figure 2). The flange area was treated with an arc osteotomy, and the radius of the arc was 60 mm for male patients and 55 mm for female patients. A Medial HTO Plate System (Olympus Thermo Biomaterials Corp., Tokyo, Japan) and HOLLYX CCS (HOLLYX, Shizuoka, Japan) were used as implants. The β‐tricalcium phosphate (Olympus Thermo Biomaterials Corp., Tokyo, Japan) was inserted to fill the wedge gap. For postoperative therapy, immobilisation in full extension with knee bracing and unloading was conducted on postoperative Day 7, and range‐of‐motion training and partial loading were initiated from postoperative 8 day. Loading was applied increasingly for a week, with full loading at postoperative Week 4. In patients who underwent extensive meniscal suturing, unloading and immobilisation were performed for 2 weeks postoperatively.

### Radiographical measurements

**Figure 3:**
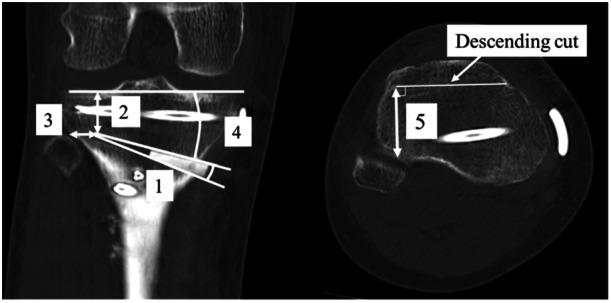
Coronal and axial computed tomography 2 weeks postoperatively. (1) Angle of opening wedge; (2) distance between hinge and the tibiofemoral articular surface; (3) distance between hinge and lateral margin of tibia; (4) angle of osteotomy between the tibiofemoral articular surface and osteotomy line; (5) anterior‐posterior width of hinge.

**Figure 4:**
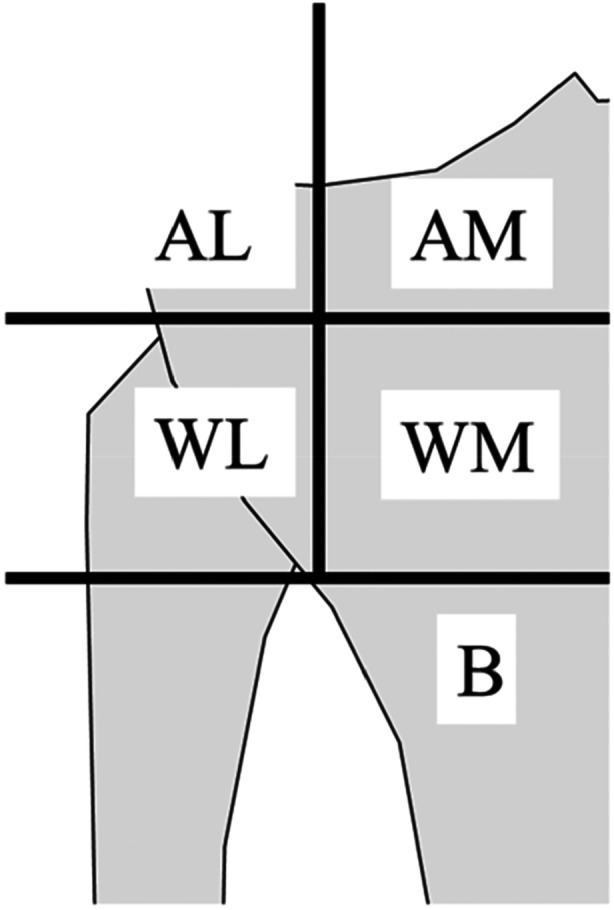
Classification of the hinge position relative to the PTFJ into five categories according to Nakamura et al. [16]. A/W/B, above/within/below the PTFJ; M/L, medial/lateral to the medial margin of the PTFJ; PTFJ, proximal tibiofibular joint.

On X‐ray the percentages of the mechanical axis (%MA) and mechanical medial proximal tibial angle (mMPTA) were measured preoperatively and 3 months postoperatively. %MA was defined as the ratio that the distance from the medial tibial plateau to the intersection of the mechanical axis was divided by the total length of the tibial plateau. mMPTA was defined as the angle between the mechanical axis and the medial tibial plateau. At the level of proximal tibiofibular joint (PTFJ), hinge fractures were investigated on coronal CT images at 2 weeks postoperatively by measuring (1) the angle of the opening wedge, (2) the distance between the hinge and the tibiofemoral articular surface, (3) the distance between the hinge and lateral margin of the tibia, and (4) the angle of osteotomy between the tibiofemoral articular surface and the osteotomy line (Figure 3). The anteroposterior width of the hinge was measured perpendicular to the descending cut of the flange on axial CT images at the proximal level of the PTFJ (Figure 3). To ensure a correct anterior‐posterior width, it is important that the descending osteotomy does not move from the anteromedial to the posterolateral direction [1]. Nakamura et al. classified the hinge position into five categories: AM, AL, WM, WL, and B (Figure 4) [16], with A, W, and B defined as Above, Within and Below the PTFJ, respectively; and M and L defined as Medial and Lateral margins of the PTFJ, respectively.

Hinge fractures were examined based on the study by Takeuchi et al. [25]. Each fracture was categorised into one of the following types: type I, a fracture within the PTFJ; type II, a fracture that reaches the distal portion of the PTFJ; and type III, a lateral plateau fracture.

### Statistical analysis

The inter‐class correlation coefficient (ICC) was used to assess inter‐rater and intra‐rater reliability for measured angles and distances on CT. Values < 0.5 were considered to have poor reliability, those between 0.5 and 0.75 were considered to have moderate reliability, those between 0.75 and 0.9 were considered to have good reliability, and those > 0.90 were considered to have excellent reliability [11].

Continuous variables are described as average ± standard deviation. A post hoc power analysis for the student's *t*‐test was performed by G*Power using the ratio of the anterior‐posterior widths between the hinge and non‐hinge fracture groups, using an effect size of 0.90 and an α error probability as 0.05. The calculated power was 0.81, indicating a sufficient sample size [4]. All statistical analyses were performed using easy R (EZR, Jichi Medical University, Saitama, Japan) running on R (R Foundation for Statistical Computing, Vienna, Austria) [9]. The normal distribution of variables was confirmed through Shaphiro–Wilk test. All variables except the value of Angle of opening wedge revealed the normal distribution. Fisher's exact test, Student's *t*‐test and the Mann–Whitney test were used for the statistical analyses, and a *p*‐value < 0.05 was considered statistically significant. Receiver operating characteristic (ROC) curve analysis was used to investigate the relationship between the anteroposterior widths of the hinge and non‐hinge fracture groups. The maximum value of Youden's index (sensitivity + specificity − 1) was used as the cut off value.

## RESULTS

### Fractures

No cases had the hinge positioned above the PTFJ, 41 cases were within the PTFJ, and one case was below the PTFJ. Of the 41 cases within the PTFJ, 21 were lateral to the medial margin of the PTFJ (Group WL) and 20 were medial to the medial margin of the PTFJ (Group WM) (Table 1). Hinge fractures were observed in 10 (23.8%) patients. The 10 hinge fracture cases were classified according to their hinge positions: six cases in Group WL and four cases in Group WM. According to the Takeuchi classification, seven cases were type I (Group WL: four cases, Group WM: three cases) and three cases were type II (Group WL: two cases, Group WM: one case), with no cases with type III fractures.

**Table 1:** Relationship between hinge position and hinge fracture.

	Non‐hinge fracture	Hinge fracture	*p*‐value
AL	0	0	—
AM	0	0	—
WL	15	6	0.72
WM	16	4	
B	1	0	—
Total	32	10	—

Abbreviations: A/W/B, above/within/below the proximal tibiofibular joint; M/L, medial/lateral to the medial margin of the proximal tibiofibular joint.

Bone union was obtained in all cases including the cases of hinge fracture. All implants were electively removed after confirming radiographic bone union, and no implant‐related complications were observed.

### Radiographical measurements

The detailed ICC values for each measurement are in Table 2. Overall, good‐to‐excellent inter‐rater agreement and good‐to‐excellent ICC values for intra‐rater reliability were obtained.

**Table 2:** Interclass correlation coefficients (ICC) values of the intra‐rater and inter‐rater reliability tests for each measurement.

	Intra‐rater	Inter‐rater
(1) Angle of opening wedge (°)	0.83 (0.71–0.91)	0.84 (0.73–0.91)
(2) Distance between hinge and the tibial articular surface (mm)	0.89 (0.80–0.94)	0.89 (0.81–0.94)
(3) Distance between hinge and lateral edge of tibia (mm)	0.94 (0.89–0.97)	0.85 (0.74–0.92)
(4) Angle of osteotomy between the tibial articular surface and osteotomy line (°)	0.82 (0.69–0.90)	0.90 (0.82–0.95)
(5) Anterior‐posterior width of hinge (mm)	0.96 (0.93–0.98)	0.96 (0.92–0.98)

*Note*: Data are shown as value (95% confidence interval).

Table 3 shows the %MA and mMPTA measurements on the preoperative and postoperative radiographs. In both the hinge and non‐hinge fracture groups, %MA and mMPTA significantly increased postoperatively (all *p* ≤ 0.01). There were no statistically significant differences in preoperative or postoperative alignment parameters (%MA, mMPTA) between the hinge and non‐hinge fracture groups.

**Table 3:** Preoperative and postoperative measurements of %MA and mMPTA on radiographs.

	Non‐hinge fracture	Hinge fracture	*p*‐value
Preoperative %MA (%)	28.6 ± 9.8 (16,41)	28.9 ± 9.1 (18, 44)	0.93
Postoperative %MA (%)	57.2 ± 7.5 (51, 67)	56.7 ± 8.5 (45, 75)	0.87
Preoperative mMPTA (°)	83.9 ± 2.2 (79, 88)	83.7 ± 2.0 (79, 86)	0.49
Postoperative mMPTA (°)	90.3 ± 2.0 (86, 94)	89.4 ± 2.1 (85, 93)	0.51

*Note*: Data are expressed as mean ± standard deviation, (minimum and maximum).

Abbreviations: mMPTA, mechanical medial proximal tibial angle; %MA, percentage of mechanical axis.

Table 4 shows the comparison of the distances measured on CT between non‐hinge and hinge fractures. No significant difference was found in the incidence of hinge fractures between the WM and WL groups. In addition, no significant differences were observed between the non‐hinge and hinge fracture groups in terms of opening wedge angles (*p* = 0.41), distances between hinge and tibial articular surface (*p* = 0.06), distances between hinge and lateral edge of tibia (*p* = 0.12), and angles of osteotomy between the tibial articular surface and osteotomy line (*p* = 0.95). However, the anterior‐posterior width of the hinge was significantly shorter in the hinge fracture group (*p* = 0.03). Table 5 shows the comparison of the distances measured on CT between the WL and WM groups. The distance between the hinge and the lateral edge of tibia and the anterior‐posterior width of the hinge were significantly different between WL and WM groups.

**Table 4:** Angles and distances in non‐hinge and hinge fractures measured on CT.

	Non‐hinge fracture	Hinge fracture	*p*‐value
(1) Angle of opening wedge (°)	7.0 ± 1.8 (4.0, 9.0)	7.2 ± 1.4 (5.0, 9.0)	0.75
(2) Distance between hinge and the tibial articular surface (mm)	18.9 ± 2.0 (15.1, 22.7)	20.4 ± 2.2 (18.4, 25.2)	0.06
(3) Distance between hinge and lateral edge of tibia (mm)	9.0 ± 3.1 (3.7, 18.2)	7.0 ± 3.9 (2.4, 13.9)	0.12
(4) Angle of osteotomy between the tibial articular surface and osteotomy line (°)	14.9 ± 3.1 (9.0, 22.0)	14.8 ± 3.5 (10.0, 20.0)	0.95
(5) Anterior‐posterior width of hinge (mm)	28.6 ± 7.3 (15.4, 46.0)	22.8 ± 4.9 (17.2, 31.9)	**0.03**

*Note*: Data are presented as mean ± standard deviation (minimum and maximum).

Abbreviation: CT, computed tomography.

**Table 5:** Angles and distances of WL and WM groups measured on CT.

	WL	WM	*p*‐value
(1) Angle of opening wedge (°)	7.1 ± 1.6 (4.0, 10.0)	7.0 ± 1.8 (4.0, 10.0)	0.87
(2) Distance between hinge and the tibial articular surface (mm)	19.0 ± 2.1 (15.2, 22.4)	19.5 ± 2.2 (16.7, 25.2)	0.52
(3) Distance between hinge and lateral edge of tibia (mm)	5.9 ± 2.2 (2.4, 10.2)	11.2 ± 2.2 (8.4, 18.2)	≤**0.01**
(4) Angle of osteotomy between the tibial articular surface and osteotomy line (°)	14.5 ± 3.2 (9.0, 22.0)	15.1 ± 3.2 (12.0, 20.0)	0.54
(5) Anterior‐posterior width of hinge (mm)	24.2 ± 5.6 (15.4, 33.7)	30.6 ± 7.3 (23.6, 46.0)	≤**0.01**

*Note*: Data are presented as mean ± standard deviation (minimum and maximum).

Abbreviations: CT, computed tomography; WL, lateral to the medial margin of the proximal tibiofibular joint; WM, medial to the medial margin of the proximal tibiofibular joint.

**Figure 5:**
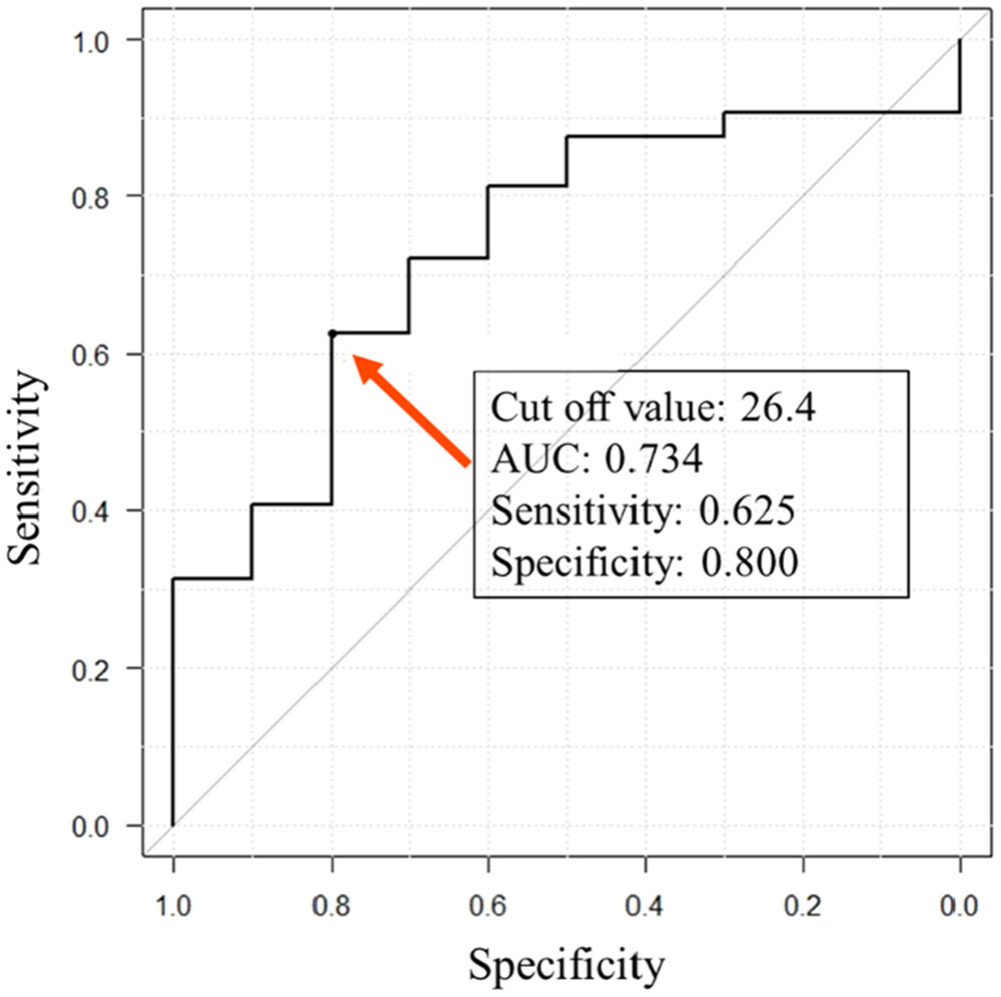
Receiver operating characteristic curve analysis was performed to investigate how different anterior‐posterior widths affect hinge fractures. AUC, area under the curve.

ROC analysis showed that Youden's index cut‐off was an anterior‐posterior width ≤ 26.4 mm with an area under the ROC curve of 0.734. The sensitivity and specificity were 62.5% and 80.0%, respectively (Figure 5).

## DISCUSSION

The most important findings of our cohort are that no type III (intraarticular) fractures were observed in cases where the hinge was placed within the PTFJ and that the anterior‐posterior width of the hinge was significantly shorter in the hinge fracture group. It is also noteworthy that this study focused on the relationship hinge fracture and hinge position in owDTO not in conventional owHTO.

In our study, the hinge fracture rate of owDTO was comparable to that in previous reports on owHTO [10]. Nakamura et al. reported that AL and AM hinge fractures tended to extend to the joint (type Ⅲ) [16]. Ogawa et al. also suggested that fractures are more likely to reach the intra‐articular surface when the hinge position is more proximal than the PTFJ [19]. In our study there were no AL and AM fractures and or type III hinge fractures. The results of our study revealed that the hinge position within the PTFJ may prevent type III fractures.

When determining the hinge position intraoperatively in our institution, the osteotomy line is determined so that the maximum length screw possible (60 mm) can be inserted into the D‐hole of the plate (Figure 1). Nha et al. reported 12 cases of implant failure around D‐hole [18]. The risk of implant failure around the D‐hole was considered to be higher in the case of hinge fractures occurring. Chen et al. also revealed inserting a screw in the D‐hole reduces stress concentration when using a double plate for a hinge fracture by finite element analysis [3]. Thus, the D‐hole is important for supporting the osteotomy area. In order to avoid stress concentration, the maximum length screw possible was inserted into the D‐hole in our institution even if hinge fracture did not happen.

The hinge position would be set to distal in order to insert a long screw into the D‐hole. No cases with hinge position AM or AL were observed in this study. While the hinge position within the PTFJ may prevent type III fractures, the possibility of a higher incidence of type II fractures might be feared. In our research three cases of type II fractures were observed, which is comparable to the incidence in other studies [13, 14]. Hinge positions that are too low, even within the PTFJ, could cause the possibility of the type II fracture. Our results showed no occurrence of type III fractures within the PTFJ but do not suggest that hinge placement within the PTFJ universally prevents all hinge‐related complications. Further research should be needed to avoid both types II and III fractures.

Moreover, Azoti et al. researched the appropriate hinge position based on the classification of Nakamura et al. by finite element analysis [2, 16]. This research showed that AM area had the lowest local stress concentration. However, the characteristics of implants make it difficult to locate hinge positions in AM area. The next lowest stresses were in the WM and WL areas. Considering that a possible longer screw can be inserted in the D‐hole, it may be also appropriate to place a hinge position within the PTFJ from this point of view. On the other hand, Kung et al. reported that the hinge at the point where the upper end of the PTFJ is located provided the lowest von mises stress, and the hinge at the point where the more distal located provided higher stress [12]. Although the appropriate hinge position remains controversial in biomechanical studies, it would be important to place a possible longer screw in the D‐hole to secure it. In our study, the hinge position was placed in the WM and WL regions for the above reasons, and the WM and WL regions might be appropriate hinge positions in owDTO because the hinge fracture rate is comparable to that of owHTO [13, 14].

In our study, the anterior‐posterior width was detected significantly shorter in the hinge fracture group. Therefore, the results of this study indicate that preservation of the antero‐posterior width of the hinge may reduce hinge fractures, and the anterior‐posterior width should be considered during osteotomy. Akiyama et al. mentioned that the descending osteotomy should avoid moving from the anteromedial to the posterolateral direction to keep the antero‐posteior width [1]. Ogawa et al. reported that a model with a thinner osteotomy of the anterior flange had the highest load resistance in the longitudinal direction at the hinge [21]. The thinner anterior flange facilitated securing the width of the anterior‐posterior hinge. If the anterior flange is thinly cut, the possibility of fracture at the same area increases. However, according to Ogawa et al. tibial tubercle fracture did not affect the clinical outcome and bony union in owDTO [20]. The anterior‐posterior width of the WL group was significantly shorter than that of the WM group (Table 5). The anterior‐posterior and medial‐lateral lengths decreased in the axial plane of the tibia as it became more distal to the knee joint [17]. Therefore, the width of the anterior‐posterior hinge within the PTFJ was shorter, suggesting that the WL group may show a trend towards a shorter anterior‐posterior width.

This study had several limitations. First, the sample size was small. Further studies are needed to increase the number of cases. Second, some patients could not be followed up, and we have now specified that the follow‐up rate was 84% (42/50 knees). Third, we did not specifically evaluate surgical accuracy or overcorrection. Despite these limitations, this study could reveal that no intraarticular (type III) fractures were observed when the hinge position was located within the PTFJ. Attention should also be directed toward preserving the anterior‐posterior hinge during operations to prevent hinge fractures.

## CONCLUSIONS

In our study cohort, no intraarticular (type III) fractures were observed when the hinge position was located within the PTFJ. However, type II fractures did occur. The results suggest that preserving the anterior‐posterior hinge width may contribute to reducing hinge fractures in owDTO. Further studies with larger sample sizes are necessary to validate these findings.

## AUTHOR CONTRIBUTIONS

Atsuki Tanaka was involved in the conception and design of the study, the acquisition, analysis and interpretation of the data, and writing the article. Daisuke Araki was involved in the conception and design of the study, development of the research, and writing the article. Takahiro Yamashita, Shohei Sano, Ryo Okada, Mitsuhiko Takahashi, and Yasushi Hashimoto were involved in the acquisition and interpretation of the data. All of the authors were involved in the critical revisions of the article for its important intellectual content, and they all approved the final version of the article.

## CONFLICT OF INTEREST STATEMENT

The authors declare no conflicts of interest.

## ETHICS STATEMENT

This retrospective study was performed in accordance with the Declaration of Helsinki and with approval from our institutional review board.

## Data Availability

The data generated and analysed during this study are included in this article.
